# Intraoperative Consultation-Related Documentation: A Three-Year Quality Improvement Audit

**DOI:** 10.1177/10668969251378629

**Published:** 2025-10-08

**Authors:** Moreen Haddad, Julianne Klein

**Affiliations:** 1 Department of Diagnostic and Molecular Pathology, 8664 Max Rady College of Medicine, Rady Faculty of Health Sciences, University of Manitoba, Winnipeg, Canada

**Keywords:** intraoperative consultation (IOC) documentation, quality improvement interventions, audit, pathology report accuracy, checklist, frozen sections

## Abstract

**Objectives:**

This study evaluates the effectiveness of interventions aimed at improving compliance with intraoperative consultation (IOC) documentation standards within the health system in Manitoba, Canada.

**Methods:**

A quality improvement study assessed IOC documentation quality within Shared Health Manitoba system. Audits of 60 consecutive IOC cases (specimens) annually (2021-2023) evaluated frozen section (FS) requisitions, final pathology reports, and quality assurance (QA) review entries. Interventions included education sessions, email reminders, posters, and a trial of checklist stickers, though the latter was unsuccessful. Statistical analyses assessed documentation trends.

**Results:**

Interventions led to some significant but inconsistent improvements. Overall documentation completeness improved in IOC requisitions from 2021 to 2022 (p = 0.0245, 95% CI = 3.69, 38.16) but not in 2023. QA review entries also showed significant improvement in the second audit in 2022, but not in the third audit in 2023 (2021 vs 2022: 0.0327, although the 95% CI lower bound was slightly negative (−2.55, 19.05). No overall significant improvements were observed in final report IOC documentation.

**Conclusion:**

Targeted interventions improved some IOC documentation aspects, but sustained success requires addressing systemic challenges through ongoing education and workflow optimization to enhance IOC-related documentation quality.

## Introduction

Intraoperative consultations (IOCs) are crucial for guiding surgical decision-making and rely heavily on the pathologist's accurate diagnosis of frozen sections (FS). While extensive research exists on the diagnostic precision of IOCs, there has been limited exploration of their non-diagnostic aspects particularly the documentation process. Local and international guidelines, such as those from the Pan-Canadian Quality Assurance Recommendations^1^ and the College of American Pathologists emphasize the importance of thorough documentation and other quality assurance measures in surgical pathology including during IOCs. However, compliance with these guidelines is inconsistent, and their impact on patient safety outcomes has not been fully explored.

Quality in pathology is generally defined as the generation of timely, accurate, and complete reports. During IOCs, pathologists are responsible for accurately documenting several critical details: the type and gross features of the fresh specimen, its orientation, appropriate inking of margins (when applicable), and handling processes, including the selection of sections for FS assessment. Additionally, the name of the pathologist performing the IOC, the time the diagnosis is reported, and the name of the healthcare worker receiving the diagnosis must be clearly recorded on IOC requisitions. Without these elements, verification of specimen handling and diagnostic communication becomes challenging, potentially compromising patient safety.^2^ Additionally, IOC findings are integrated into the final surgical pathology report, either via transcription, as is the case at our institutions, or direct entry into the laboratory information system (LIS) by the FS pathologist. A second pathologist responsible for signing out the final report must ensure completeness of IOC documentation in the final report, document correlations or discrepancies per institutional guidelines, and promptly communicate any frozen section/permanent section discrepancies to the clinical team to prevent patient management issues.^1,2^

The accuracy of the final diagnosis is dependent on the effective execution of these steps. Peer review plays a pivotal role in ensuring the quality of reports and safeguarding patient care.^3^ Review of report defects is an important component of a laboratory's quality management plan, as this activity enables the laboratory to detect weakness in workflow and organization, identifying opportunities for targeted interventions aimed at reducing defects. Studying report defects is a strong quality measure since it allows detection of problems in all aspects of the diagnostic process.^4^ At our local institutions, we have established IOC documentation guidelines aligning with the Pan-Canadian Quality Assurance Recommendations for Interpretive Pathology to promote best practices and enhance quality assurance.^1^ Regular audits are performed to assess the completeness and accuracy of IOC reports details. Despite these efforts, inconsistencies in documentation persist, highlighting the need for targeted interventions. To address these challenges, we conducted a quality improvement project to identify areas needing improvement and implement measures to enhance compliance with documentation standards across hospitals under Shared Health, a provincial health organization in Manitoba that integrates services across multiple sites.

The workflow in our FS laboratories is standardized, with shared guidelines established by Shared Health Manitoba. Across our health system, FS services are provided at five sites, with each site having dedicated pathology staff responsible for IOCs during regular working hours. After hours and on weekends, FS requests are primarily handled at two of these sites. During these periods, a single on-call pathologist is responsible for both sites; however, the overall FS case volume is typically low, and although simultaneous FS requests from both sites are possible, such instances are uncommon. For the purposes of this study, a “case” refers to the set of specimens submitted for IOC from a single patient during one surgical procedure.

Although our system has implemented standardized FS documentation guidelines across multiple sites, we recognize that institutional practices around IOCs vary widely. Differences in staffing models, available resources, LIS platforms, and case volumes can result in significant variability in how FS services are delivered and documented. Despite this heterogeneity, the core principles of IOC, including accurate diagnosis, timely communication, and complete documentation, remain consistent. This underscores the importance of promoting shared documentation standards that can be adapted to local workflows. Our study reflects an effort to implement such standardization within a multi-site system, and may offer insights applicable to other institutions navigating similar challenges.

FS specimens are accompanied by a requisition form that includes a designated space for IOC documentation. Specimens are typically transported from the operating room (OR) to the FS suite by OR staff or a hospital porter. In some instances, the operating surgeon, a fellow, or a resident may deliver the specimen personally, particularly when specific orientation or handling instructions must be conveyed directly. The same requisition form is used to submit multiple specimen parts, including more than one FS when requested. Pathologists handwrite the diagnosis and other IOC details directly on the requisition, which is later transcribed by administrative staff into our pathology-specific LIS system, CoPath. This information is ultimately integrated into the final pathology report. Pathologists responsible for final sign-out must ensure the accuracy of the IOC documentation, including performing a QA review for correlation within CoPath that should reflect the number of discreet reports provided to the surgeon. This is done using a standardized QA Diagnosis Review module. This entry includes predefined classification options such as “Correlation – IOC,” “Discordance,” or “Deferral” and mandates additional documentation for discrepancies, including surgeon communication and potential clinical impact. This protocol ensures standardized classification of discrepancies across all audited cases.

Ideally, the pathologist who performs the IOC should not be the same individual who signs out the final diagnosis.^5^ This allows for an independent review of findings, promoting quality assurance and minimizing potential cognitive bias. While this approach is considered best practice in quality programs, it may not always be feasible in institutions with limited staffing. In such cases, alternative quality safeguards such as QA reviews entered by a second pathologist (as required by our local institutional guidelines), peer review of selected cases, or regular audits can serve as effective substitutes to uphold diagnostic standards.

Despite standardized workflows and institutional guidelines, documentation practices across sites remained inconsistent, highlighting the need for targeted improvement efforts. To improve compliance with these processes, we implemented targeted interventions such as teaching sessions, personalized reminders, posters, and a trial introduction of a checklist sticker. This study evaluates the effectiveness of these interventions in improving adherence to documentation standards in original frozen section requisitions, final pathology reports, and QA reviews entered into CoPath, with the overarching goal of ensuring the reliability and accuracy of intraoperative consultations.

## Methods

We audited 60 consecutive IOC cases from each of the years 2021, 2022, and 2023. These cases represented a diverse selection from various surgical pathology specialties, including neuropathology, and encompassed specimens from all hospitals within Shared Health system. Subspecialty-trained pathologists cover neuropathology IOCs during regular working hours. However, after hours and on weekends, these and all other IOCs are managed by the on-call surgical pathologist, consistent with our general coverage model.

The consecutive cases were selected by a member of the quality assurance (QA) team. A pathology resident manually extracted data from CoPath, focusing on original FS requisitions, final signed-out reports, and QA entries for these cases.

**Figure 1.**
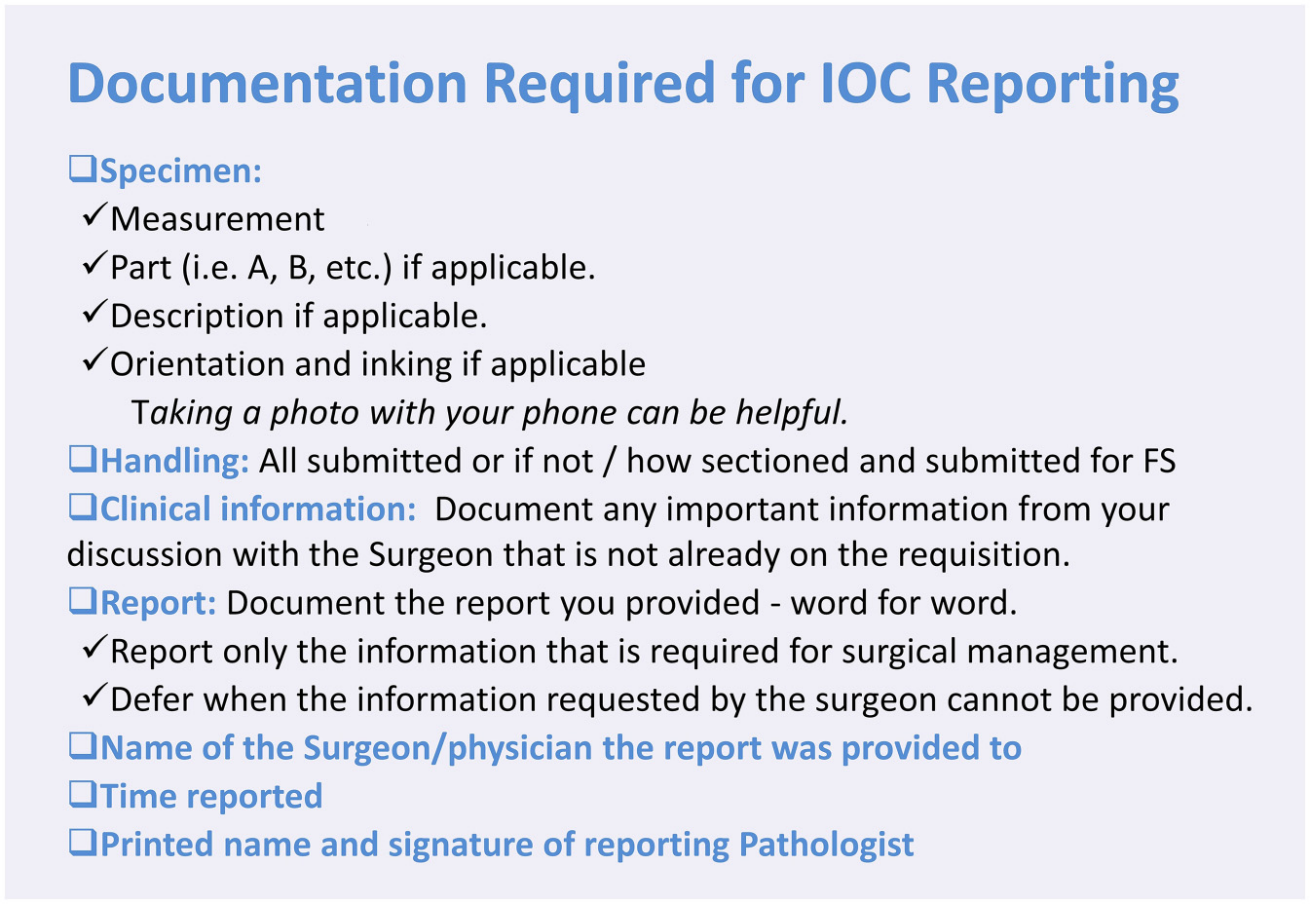
Posters were emailed to pathologists and displayed in FS areas.

Data collection was standardized using a pre-constructed electronic form, which was subsequently entered into an Excel sheet for analysis. We calculated the percentage of complete entries for each documentation category and generated visual graphs for representation. To assess statistical significance, Python was used to conduct a two-proportion Z-test for year-to-year comparisons of each category on the standard form and a one-tailed paired t-test for overall dataset comparisons across years, including FS requisitions, final reports, and QA review entries. The Python code for statistical analysis was generated with the assistance of ChatGPT (OpenAI, 2025). A 95% confidence interval (CI) was calculated for all estimates, and a p-value ≤ 0.05 was considered statistically significant. The selection of statistical tests used in this study was guided by recommendations from the University of Manitoba biostatistics consultation service. The initial audit, conducted in 2021, identified gaps in compliance. The results were presented during QA Grand Rounds to provide education and raise awareness. As part of the intervention, posters summarizing IOC documentation guidelines were distributed via email to pathologists and displayed in frozen section areas across hospitals (Figure 1).

**Figure 2.**
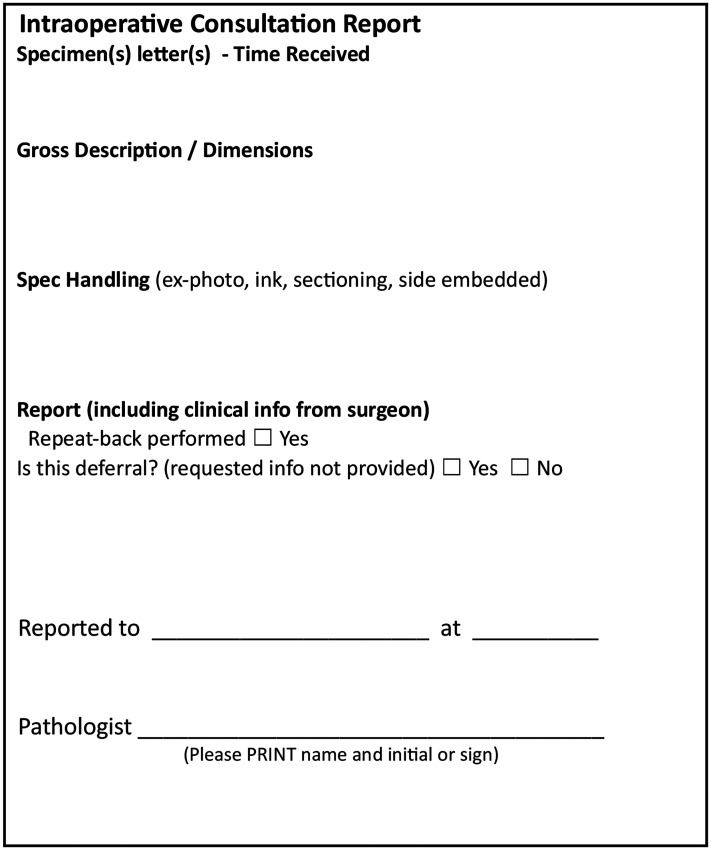
Intraoperative consultation checklist sticker.

In 2022, a second audit was performed, and the findings were again shared during QA Grand Rounds. To further address gaps, an optional use of a checklist sticker was introduced for IOC documentation (Figure 2). The sticker goal was to provide a structured checklist to remind pathologists of required documentation elements and to address the limited space on requisitions. Additional personalized reminders were sent to pathologists to reinforce QA review compliance within CoPath.

A third audit, conducted in 2023, evaluated the impact of these interventions. Ethics approval for this study was obtained from the University of Manitoba Health Research Ethics Board (HREB).

This study is reported in accordance with the Standards for Quality Improvement Reporting Excellence (SQUIRE) guidelines to ensure the systematic and transparent reporting of quality improvement interventions (SQUIRE 2.0 Guidelines).^6^

## Results

### Intraoperative Reports Documentation Categories

**Figure 3.**
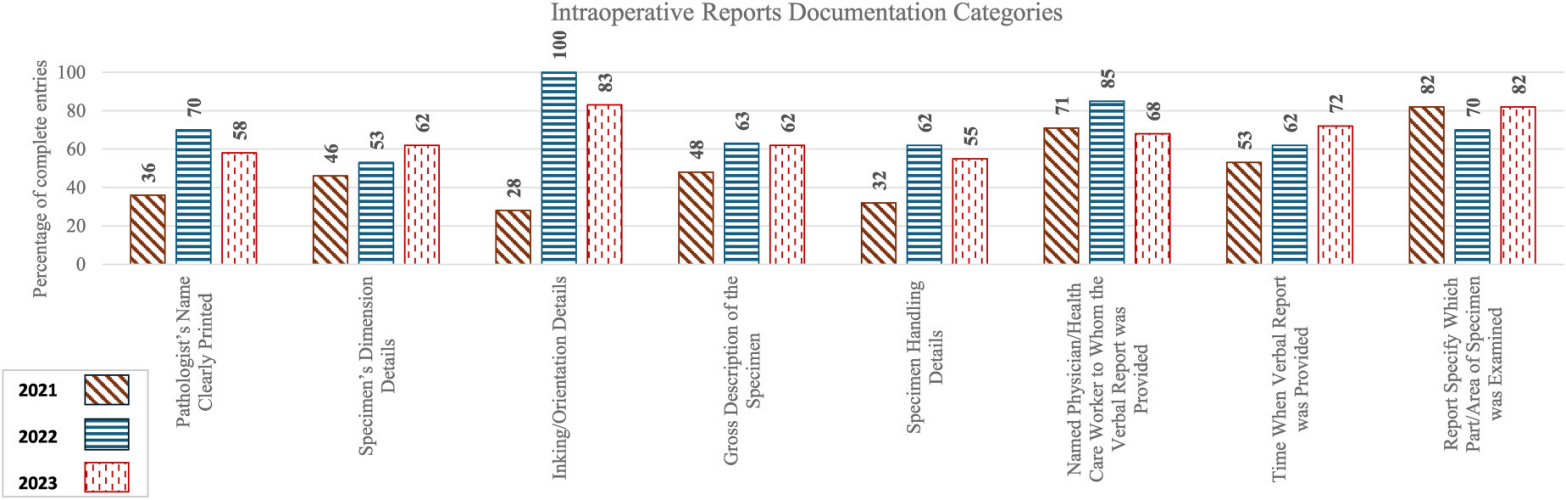
Percentage of complete entries of individual categories in the intraoperative consultation requisition.

Table 1 and Figure 3 summarize the percentage of satisfactory entries for each documentation category in the original FS requisition along with the statistical significance of year-to-year changes.

**Table 1.** Percentage of complete entries and year-to-year statistical significance in intraoperative consultation requisition documentation.

Audit Section	Category	Percentage of complete entries in 2021	Percentage of complete entries in 2022	Percentage of complete entries in 2023	Z-test (2021 vs 2022) for individual category statistical significance	Z-test (2022 vs 2023) for individual category statistical significance	t-test (2021 vs 2022) for overall statistical significance	t-test (2022 vs 2023) for overall statistical significance
**Intraoperative Reports Documentation**							**p** **=** **0.025** (3.7, 38.2)	p = 0.748 (−11.1, 5.2)
	Pathologist's Name Clearly Printed	36	70	58	**p** **=** **0.015** (−62.0, −6.6)	p = 0.408 (−16.0, 39.4)	-	-
	Specimen's Dimension Details	46	53	62	p = 0.606 (−35.0, 20.4)	p = 0.557 (−36.0, 19.4)	-	-
	Inking/Orientation Details	29	100	83	**p** **<** **0.001** (−99.2, −43.8)	p = 0.238 (−11.0, 44.4)	-	-
	Gross Description of the Specimen	48	63	62	p = 0.286 (−42.8, 12.6)	p = 0.904 (−26.0, 29.4)	-	-
	Specimen Handling Details	32	62	55	**p** **=** **0.037** (−57.2, −1.8)	p = 0.641 (−21.1, 34.3)	-	-
	Named Physician/Health Care Worker to Whom the Verbal Report was Provided	71	85	68	p = 0.336 (−41.3, 14.1)	p = 0.238 (−11.0, 44.4)	-	-
	Time When Verbal Report was Provided	54	62	72	p = 0.567 (−35.8, 19.6)	p = 0.480 (−37.7, 17.7)	-	-
	Report Specifies Which Part/Area of Specimen was Examined	82	70	82	p = 0.396 (−15.7, 39.7)	p = 0.412 (−39.3, 16.1)	-	-

IOC = Intraoperative Consultation, QA = Quality Assurance

The overall 2021–2022 improvement was statistically significant (p = 0.0245, 95% CI = 3.69, 38.16), but the overall 2022–2023 change was not.

Improvements in documentation of most individual categories were noted during the second audit in 2022 compared to the initial audit in 2021. However, the third audit in 2023 revealed a decline in many categories that previously showed improvement. The trial checklist sticker, introduced as an optional tool in 2022, was not adopted by pathologists, limiting its potential impact.

1. Is the Pathologist's Name Clearly Printed on IOC Requisition?

Documentation improved significantly from 2021 (36%) to 2022 (70%) (p = 0.0153, 95% CI = -62.02, −6.58). However, a decline to 58% was observed in 2023. Issues included missing names and illegible handwriting.

2. Are Specimen's Dimension Details Written on the Requisition?

Documentation improved steadily: 46% (2021), 53% (2022), and 62% (2023), though differences were not statistically significant.

3. Are Inking/Orientation Details Written on the Requisition, When Applicable?

Significant improvement was observed between 2021 (29%) and 2022 (100%) (p < 0.00001, 95% CI = -99.22, −43.78). However, a decline to 83% in 2023 was noted.

4. Is a Gross Description of the Specimen Documented?

Documentation rose from 48% in 2021 to 63% in 2022 and further to 70% in 2023, although year-to-year changes were not statistically significant.

5. Is the Specimen Handling Details Documented (ie Sections Selection for FS and Whether Entirely Submitted)?

Documentation improved significantly from 32% in 2021 to 62% in 2022 (p = 0.0370, 95% CI = -57.22, −1.78). However, a decline to 55% in 2023 was noted.

6. Is There a Named Physician/Health Care Worker to Whom the Verbal Report was Provided?

Documentation improved from 2021 (71%) to 2022 (85%), although not statistically significant. A decline to 68% was noted in 2023.

7. Is There a Time When Verbal Report was Provided?

Gradual improvement was noted from 54% (2021) to 62% (2022), and 72% (2023), however, there were no significant differences between years.

8. Does the Report Specify Which Part/Area of Specimen was Examined:

Documentation declined from 82% in 2021 to 70% in 2022 before improving back to 82% in 2023. Changes were not statistically significant.

### Final Reports IOC Section Documentation Categories

**Figure 4.**
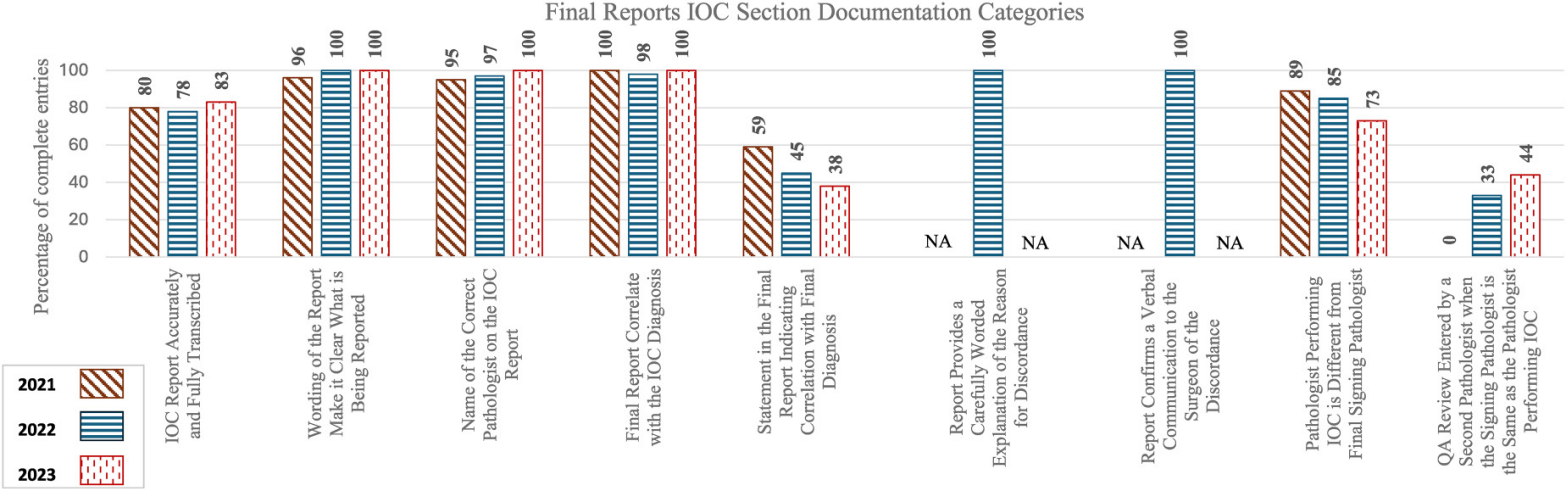
Percentage of complete entries of individual categories in the intraoperative consultation section of final report.

Table 2 and Figure 4 summarize the percentage of satisfactory entries for each documentation category in the IOC section of the final report along with the statistical significance of year-to-year changes.

**Table 2.** Percentage of complete entries and year-to-year statistical significance in intraoperative consultation section of final report documentation.

Audit Section	Category	Percentage of complete entries in 2021	Percentage of complete entries in 2022	Percentage of complete entries in 2023	Z-test (2021 vs 2022) for individual category statistical significance	Z-test (2022 vs 2023) for individual category statistical significance	t-test (2021 vs 2022) for overall statistical significance	t-test (2022 vs 2023) for overall statistical significance
**Final Reports IOC Section Documentation**							p = 0.663 (−8.3, 13.2)	p = 0.546 (−5.1, 5.8)
	IOC Report Accurately and Fully Transcribed	80	78	83	p = 0.904 (−26.0, 29.4)	p = 0.724 (−32.7, 22.7)		
	Wording of the Report Make it Clear What is Being Reported	96	100	100	p = 0.799 (−31.3, 24.1)	p = 1.000 (−27.7, 27.7)	-	-
	Name of the Correct Pathologist on the IOC Report	95	97	100	p = 0.887 (−29.7, 25.7)	p = 0.810 (−31.1, 24.3)	-	-
	Final Report Correlate with the IOC Diagnosis	100	98	100	p = 0.904 (−26.0, 29.4)	p = 0.904 (−29.4, 26.0)	-	-
	Statement in the Final Report Indicating Correlation with Final Diagnosis	59	45	38	p = 0.326 (−13.8, 41.6)	p = 0.636 (−21.0, 34.4)	-	-
	Pathologist Performing IOC is Different from Final Signing Pathologist	89	85	73	p = 0.766 (−23.5, 31.9)	p = 0.408 (−16.0, 39.4)	-	-
	QA Review Entered by a Second Pathologist	0	33	44	**p** **=** **0.020** (−60.7, −5.3)	p = 0.449 (−38.4, 17.0)	-	-

IOC = Intraoperative Consultation, QA = Quality Assurance

No statistical significance was observed for the overall 2021–2022 or 2022–2023 changes. Documentation trends within the individual categories of the IOC sections of final reports showed minimal changes, with the notable exception of the inclusion of a QA note entered by a second pathologist when the FS and final signing pathologist are the same.

1. Is the IOC Report Accurately and Fully Transcribed?

Accuracy rates showed minor fluctuations: 80% (2021), 78% (2022), and 83% (2023), with no statistically significant differences. Errors were attributed to illegible handwriting, insufficient clarification of wording by the transcriptionist with pathologists, and failure to transcribe information that was written outside the designated IOC area due to inadequate space on the IOC reports.

2. Does the Wording of the Report Make it Clear What is Being Reported, in Particular if the Word “Deferral” Appears in the Report?

Clarity remained high across all years, increasing from 96% (2021) to 100% (2022), with a slight decline to 98% (2023). Differences were not statistically significant.

3. Is the Name of the Correct Pathologist on the IOC Report?

Accuracy slightly increased from 95% (2021) to 97% (2022) and reached 100% in 2023, with no statistical significance.

4. Does the Final Report Correlate with the IOC Diagnosis?

Correlation remained consistently high: 100% in 2021 and 2023, with a slight dip to 98% in 2022 due to a single case with discrepancy, with no statistical significance.

5. Is there a Statement in the Final Report Indicating Correlation with Final Diagnosis?

Documentation rates declined steadily from 59% in 2021 to 45% in 2022, and 38% in 2023, but changes were not statistically significant.

6. If there is a Discordance, Does the Report Provide a Carefully Worded Explanation of the Reason?

For the single discordant case in 2022, a detailed explanation was documented.

7. If there is a Discordance, Does the Report Confirm a Verbal Communication to the Surgeon?

For the single discordant case in 2022, a verbal communication to the surgeon was documented.

8. Is the Pathologist Performing IOC Different from Final Signing Pathologist?

Rates slightly declined from 89% in 2021 to 85% in 2022 and 73% in 2023, with no statistical significance.

9. Is There a QA Review Entered by a Second Pathologist when the Signing Pathologist is the Same as the Pathologist Performing IOC?

QA reviews entered by a second pathologist in these cases increased significantly from 0% in 2021 to 33% in 2022 (p = 0.0196, 95% CI = -60.72, −5.28) and further to 44% in 2023, although not statistically significant.

### Correlation-Related CoPath QA Review Categories

**Figure 5.**
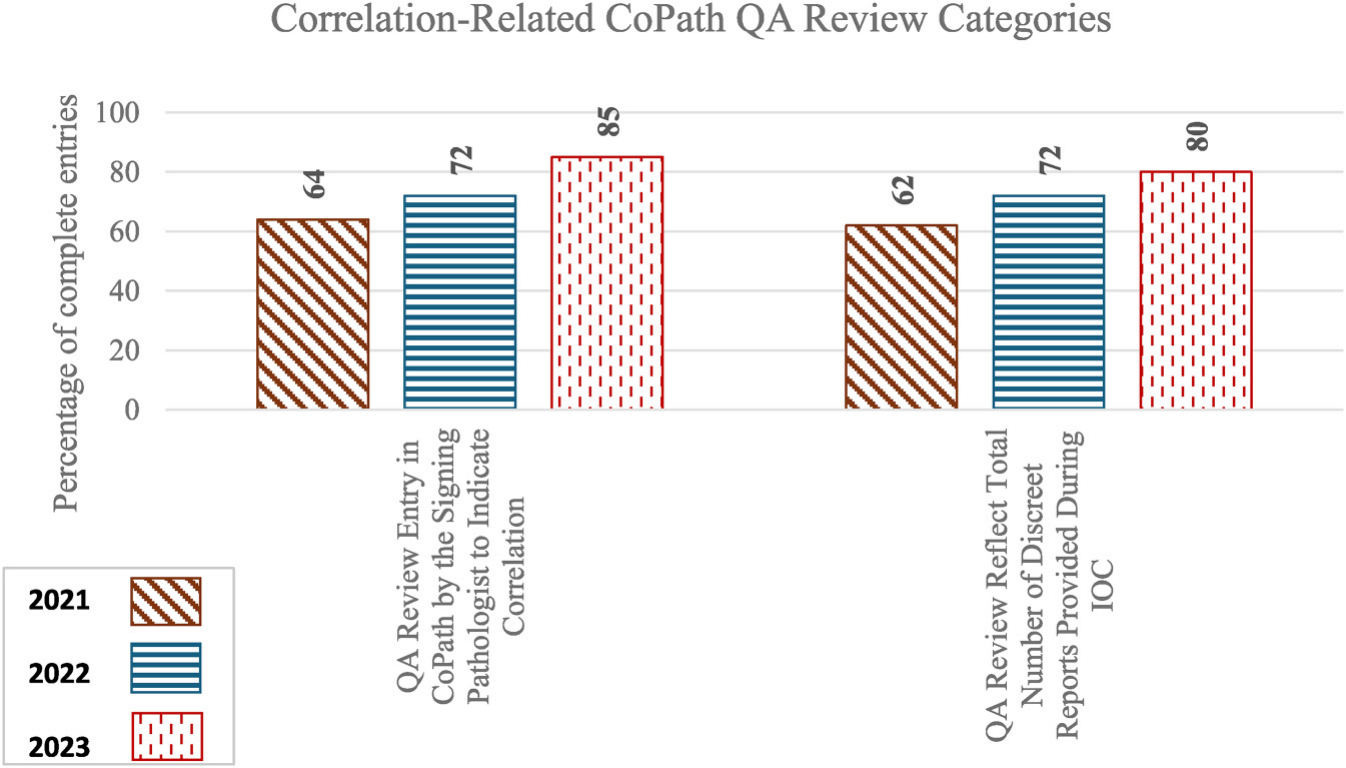
Percentage of complete entries of individual categories in CoPath QA entries.

Table 3 and Figure 5 summarize the percentage of satisfactory entries for each documentation category in CoPath QA review along with the statistical significance of year-to-year changes.

**Table 3.** Percentage of complete entries and year-to-year statistical significance of CoPath QA entries.

Audit Section	Category	Percentage of complete entries in 2021	Percentage of complete entries in 2022	Percentage of complete entries in 2023	Z-test (2021 vs 2022) for individual category statistical significance	Z-test (2022 vs 2023) for individual category statistical significance	t-test (2021 vs 2022) for overall statistical significance	t-test (2022 vs 2023) for overall statistical significance
**Correlation-related CoPath QA Review**							**p** **=** **0.0327** (−2.55, 19.05)	p = 0.0718 (−20.87, 42.67)
	QA Review Entry in CoPath by the Signing Pathologist to Indicate Correlation	64	72	85	p = 0.601 (−35.1, 20.3)	p = 0.343 (−41.1, 14.3)		
	QA Review Reflect Total Number of Discreet Reports Provided During IOC	63	72	80	p = 0.520 (−36.8, 18.6)	p = 0.553 (−36.1, 19.3)	-	-

IOC = Intraoperative Consultation, QA = Quality Assurance.

The overall improvement from 2021 to 2022 was statistically significant (p = 0.0327), despite the lower bound of the 95% confidence interval being slightly negative (−2.55, 19.05). While there was continuous improvement, the change from 2022 to 2023 was not statistically significant (p = 0.0718, 95% CI: −20.87, 42.67). Documentation improvements across individual QA entry categories remained steady over the years.

1. Is There a QA Review Entry in CoPath by the Signing Pathologist to Indicate Correlation?

Rates increased from 64% in 2021 to 72% in 2022 and 85% in 2023, although year-to-year changes were not statistically significant.

2. Does the QA Review Reflect Total Number of Discreet Reports Provided During IOC?

Rates increased from 63% in 2021 to 72% in 2022 and 80% in 2023, although the year-to-year changes were not statistically significant.

Percentage of Complete Reports and QA Entries:

The number of cases with complete documentation in all categories increased from 0% (2021) to 10% (2022) but dropped to 3% in 2023.

## Discussion

Pathologists play a critical role in ensuring accurate and comprehensive documentation during IOCs. Our study identified inconsistencies in IOC-related documentation and implemented a multifaceted intervention strategy, including educational sessions, email reminders, posters, and a trial checklist sticker, to address these issues.

**Figure 6.**
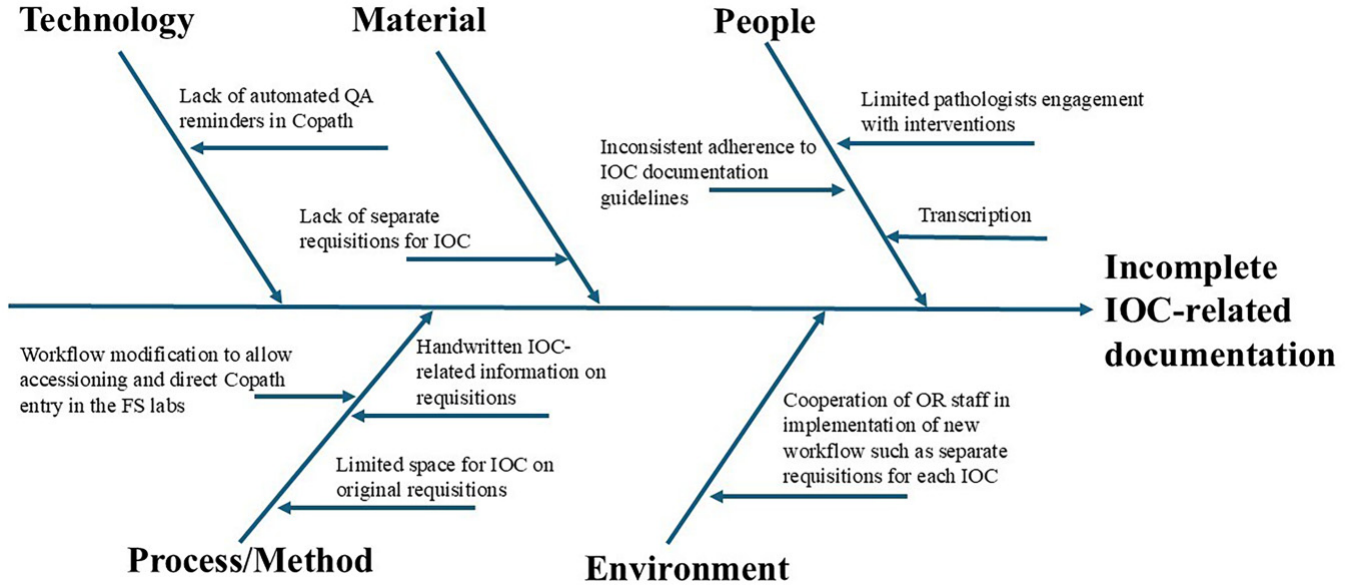
Fishbone diagram illustrating local potential causes for incomplete intraoperative consultation-related documentation.

A fishbone diagram (Figure 6) illustrates a root cause analysis highlighting potential local factors contributing to inconsistencies in IOC documentation. Due to workflow complexity, staffing challenges, and cost constraints, not all identified issues could be addressed, limiting the overall impact of our interventions. Our approach primarily targeted the “People” component through teaching, posters, and personal reminders, while the “Materials” and certain aspects of the “Process/Method” components were addressed through the trial checklist sticker.

Audits of IOC reports revealed initial improvements in documentation elements following the second audit, but sustaining these gains proved challenging in the third audit. For example, the documentation of pathologists’ names on requisitions improved from 36% in 2021 to 70% in 2022 (p = 0.0153, 95% CI = -62.02, −6.58) but subsequently declined to 58% in 2023. Documentation of inking/orientation details increased from 29% to 100% (p < 0.00001, 95% CI = -99.22, −43.78) before declining to 83% in 2023. Similarly, documentation of specimen handling details improved from 32% to 62% (p = 0.0370, 95% CI = -57.22, −1.78) but dropped to 55% in 2023. Other elements, such as specimen dimensions, gross description, the recipient of the verbal report, and the time of the verbal report, showed only non-significant improvements in the second audit and also declined in the third audit. Documentation of the specific part of the specimen examined exhibited slight year-to-year fluctuation.

Accurate documentation of specimen dimensions and gross descriptions is particularly crucial for small specimens and biopsies to prevent diagnostic errors, such as specimen contamination or mix-ups, which can impact diagnostic accuracy and patient safety.^7^ Recording the recipient of the verbal report is important for medicolegal purposes and ensures verification when needed, helping to minimize errors that could compromise patient management. Additionally, timely reporting in surgical pathology is essential, and recording the time of diagnosis aids in verifying adherence to the standard turnaround time recommended by the College of American Pathologists while facilitating FS turnaround time audits.^4,8^

Regarding the completeness of IOC documentation in final reports, no significant changes were observed overall. This is likely because the interventions focused more on pathologists performing the IOC rather than those responsible for the final sign-out of reports. The most notable improvement was seen in QA review entries when the same pathologist performed both the IOC and final pathology. QA review documentation improved significantly from 0% in 2021 to 33% in 2022 (p = 0.0196, 95% CI = -60.72, −5.28) and further to 44% in 2023. Despite these improvements, this remains an area requiring further attention. Having the same pathologist perform both the IOC and final diagnosis reduces the opportunity for peer review, which plays a critical role in identifying discrepancies that could affect patient management.

Monitoring the correlation between frozen section IOCs and final diagnoses based on permanent sections is a key component of anatomic pathology laboratory QA and is required for CAP certification. QA review entries in CoPath related to this correlation showed year-to-year improvement, particularly in the second audit (2021 vs 2022, p = 0.0327, 95% CI = -2.55, 19.05). This suggests that personalized reminders were somewhat effective in improving documentation practices.

Only one discordant diagnosis was identified during the study period. While this rate is lower than what is reported in the literature, including CAP Q-Probe data,^9^ discrepancies in our system are formally classified and documented in the LIS by the final signing pathologist or an independent reviewer, using predefined QA categories. This process requires additional documentation and clinical follow-up for any discordance or inappropriate deferral. The relatively low discrepancy rate may reflect our small sample size, and the descriptive nature of the audit, which primarily targeted documentation quality rather than diagnostic accuracy.

While structured tools, such as checklists, have been shown to enhance documentation without compromising turnaround times,^10^ our study encountered challenges in their implementation. The checklist sticker was not widely adopted by pathologists, likely due to its voluntary nature, concerns about workflow disruption, and limited perceived utility. These factors, which are also common barriers reported in other healthcare settings,^11–13^ highlight the need to address user engagement and workflow integration when introducing new tools. Such barriers reflect a broader issue: optional tools and education-based interventions, though useful in the short term, often fail to produce sustained improvement.^14^ Grand Rounds, posters, and email reminders may raise initial awareness, but their long-term impact is often undermined by staff turnover, shifting clinical demands, and cognitive overload. Newly hired pathologists may miss prior training sessions, and even engaged staff may forget content over time, especially when tools are not built into routine workflow.^13^ Behavioral science literature support this: tools requiring extra steps or disrupting established routines are frequently ignored unless tied to real-time feedback or accountability mechanisms.^11–13^

To achieve lasting improvements, quality efforts must move beyond education and incorporate system-level design. This includes embedding mandatory documentation prompts into LIS, linking checklist completion to routine audits or feedback, and integrating documentation standards into onboarding. Equally important is addressing the absence of strong motivators and consequences for noncompliance. When adherence relies solely on professional accountability, it can be deprioritized in high-pressure clinical environments. Without timely feedback, audit mechanisms, or awareness of medicolegal responsibilities, there is little incentive for consistent compliance.^12^ Future efforts should align intrinsic motivation, such as awareness of patient safety implications, with system-level supports. A combination of embedded digital tools, mandatory QA checkpoints, and structured feedback loops may help shift documentation quality from an individual obligation to an institutional standard.^15^

Handwritten requisitions posed additional challenges, including legibility and transcription errors, consistent with previous findings.^16–18^ These issues underscore the importance of transitioning to electronic documentation systems to minimize errors and enhance efficiency. Digital requisitions improve legibility, reduce transcription errors, and allow for standardized fields in IOC documentation. They also integrate more effectively with LIS and electronic health records. However, challenges such as initial implementation costs, required staff training, and ensuring immediate, reliable access in FS settings, where turnaround times are critical, must be addressed.

As pathology continues its transition to digital workflows, new tools and advancements will present opportunities for further automation, standardization, and improved documentation quality.^4^ The use of digital pathology for IOC is a promising advancement, particularly beneficial in multi-site health systems and settings with limited on-site coverage. Advantages include real-time remote access, rapid consultation with subspecialists, improved image archiving, and enhanced workflow efficiency. Recent studies have demonstrated the feasibility and diagnostic accuracy of digital pathology for frozen section reporting, supporting its potential integration into routine practice.^19^ With a validated whole-slide imaging platform already in place at our institutions, the evolution toward digital IOC documentation may further enhance consistency, accessibility, and timely communication. This study's strengths include a comprehensive, multi-year audit approach and iterative interventions aimed at improving IOC documentation. However, its limitations include the voluntary nature and lack of adoption of the checklist sticker, potential reviewer bias, an inability to address all factors identified in the root cause analysis, and the absence of data measuring the direct impact of documentation quality on patient outcomes.

Building on these findings, and to address the limitations identified, including those from the root cause analysis, future strategies should aim to sustain improvements and further enhance patient care and safety by addressing all factors identified in the fishbone analysis. This includes strengthening pathologist engagement, optimizing workflows, and integrating electronic documentation systems. By clarifying specific obstacles to sustained compliance, this project may serve as a reference point for institutions facing similar challenges. Moreover, by highlighting where voluntary or education-based approaches fall short, it offers practical insight for the development of audit mechanisms, workflow integration, and documentation tools. As quality improvement is often incremental, understanding and addressing systemic barriers remains essential for long-term success.

Although no adverse patient events directly attributable to incomplete documentation were identified during the study period, our initiative aimed to proactively strengthen practices that, if left unaddressed, could lead to communication breakdowns, diagnostic delays, or medicolegal risks over time.
